# Quality Initiative to Improve time to Antibiotics for Febrile Pediatric Patients with Potential Neutropenia

**DOI:** 10.1097/pq9.0000000000000095

**Published:** 2018-08-09

**Authors:** Kathy Monroe, Clay T. Cohen, Kimberly Whelan, Amber King, Lisa Maloney, Janet Deason, John Charles Nichols, Gregory K. Friedman, Matthew Kutny, Leslie Hayes

**Affiliations:** From the * Department of Pediatrics, Division of Emergency Medicine, Pediatric Emergency Medicine, University of Alabama, Birmingham, Ala.; † Department of Pediatrics, Division of Hematology, Section of Hematology-Oncology, Texas Children’s Cancer and Hematology Centers, Baylor College of Medicine, Houston, Tex.; ‡ Department of Pediatrics, Division of Hematology, Section of Hematology-Oncology, University of Alabama, Birmingham, Ala.; § Nursing Informatics Specialist, Department of Pediatrics, Division of Emergency Medicine, Childrens of Alabama, Birmingham, Ala.; ¶ Advanced Unit Educator, Department of Pediatrics, Division of Emergency Medicine, Childrens of Alabama, Birmingham, Ala.; ‖ Department of Pharmacy, Childrens of Alabama, Birmingham, Ala.; ** Auburn University, Auburn, Ala.; †† Department of Pediatrics, Division of Intensive Care, Pediatric Intensive Care, University of Alabama, Birmingham, Ala.

## Abstract

**Introduction:**

In patients who are immunocompromised, fever may indicate a life-threatening infection. Prompt time to antibiotic administration in febrile patients at risk for neutropenia has been identified by national and international panels as a key benchmark of quality care in emergent situations. A quality improvement initiative to improve health care provided in a pediatric emergency department (ED) is described.

**Methods:**

A clinical pathway was previously initiated in a pediatric ED with a goal of improving time to antibiotics for febrile neutropenia patients. An agreed upon pathway and order set being initiated. Improvements were seen but not to the desired level. This project involved an improvement cycle that focused on nonvalue added time in the workflow.

**Results:**

Percent of patients receiving antibiotics within the goal time of 1 hour increased from 40% to 80% with the intervention. Process measures including arrival to ED bed time, ED bed to antibiotic order time and antibiotic order time to delivery time were followed.

**Conclusion:**

Clinical guidelines, order sets and detailed understanding of the actual workflow at the point of care delivery can be instrumental in achieving the goals of reducing time to antibiotics.

## PROBLEM DESCRIPTION

A retrospective chart review of patients presenting with fever and neutropenia at the Children’s of Alabama pediatric emergency department (ED) between August 2010 and December 2011 revealed that while the average time to antibiotic administration in these patients was 63 minutes, unacceptable variation existed with only 30% of these patients receiving antibiotics within the goal time of 1 hour. A quality improvement (QI) project was chartered that addressed apparent drivers including patient identification, laboratory testing, and antibiotic ordering. In a previous report, we described the results of this QI project by which the percentage of patients meeting the 1 hour goal increased from 35% to 51%. Despite the initial improvement, the percentage of patients receiving antibiotics within 1 hour remained below the desired results. In this report, we describe our QI project that reduced non–value-added time in the workflow, which impacted multiple levels of care delivery and improved and sustained the time to delivery of antibiotics for febrile neutropenia patients presenting to the ED.

## AVAILABLE KNOWLEDGE AND RATIONALE

Neutropenia may manifest as a congenital or acquired benign hematologic process or as a serious complication of myelosuppressive treatments for cancer. When a patient’s absolute neutrophil count drops below 500/μL, fever can signal severe risk for infectious morbidity and mortality.^1^ Compared with children without cancer who develop sepsis, children with cancer and sepsis have a 1.6-fold increased risk of death.^2^ Studies indicate that over 80% of individuals with a hematologic malignancy will develop fever during at least 1 chemotherapy cycle indicating potential serious infection risk.^3^

Unfortunately, even patients who appear stable can deteriorate significantly in the resulting short interval before antibiotics are initiated. Prolonged time from arrival in an ED until antibiotic administration can result in adverse outcomes.^1^ The American Society of Oncology and an international guideline panel recommend administering the first dose of antibacterial therapy as soon as possible after triage (goal within an hour) to patients with febrile neutropenia.^4^ These recommendations have led our institution to develop clinical pathways to improve time to antibiotics for patients with febrile neutropenia.

The literature supports the prompt use of antibiotics in febrile neutropenic patients as Salstrom et al.^6^ showed that pediatric patients who receive antibiotics for fever and neutropenia in less than 60 minutes have decreased intensive care needs. The Salstrom QI study used Lean Methodology and Plan Do Study Act (PDSA) cycles to track and improve time to antibiotics and associated clinical outcomes (length of stay, duration of fever, bacteremia, intensive care unit consultation or admission, and mortality). Moreover, time to antibiotics in less than 60 minutes was associated with an 82.1% relative risk reduction for mortality when compared with those receiving antibiotics in greater than 60 minutes.^6^

## SPECIFIC AIMS

Our specific aim was to improve and maintain the percentage of febrile neutropenic patients receiving antibiotics within 1 hour of arrival in the ED. Additional aims were to improve process measures of (1) time from triage to placement in an ED room to 10 minutes; (2) time from placement in ED room to antibiotic order to 25 minutes; and (3) time from antibiotic order to administration goal: 25 minutes.

## METHODS

### Context

This initiative was conducted at the Children’s of Alabama Emergency Department. The hospital is a free-standing pediatric center located in an urban setting. The ED sees an annual volume of over 70,000 patients. The ED sees 160–170 patients with fever and potential neutropenia per year. Approval for this research was obtained from the Institutional Review Board of The University of Alabama Birmingham.

### Intervention

A QI project was performed involving a multidisciplinary team (hematology/oncology, emergency medicine, pharmacy, hospital nursing). This team reviewed cases in detail and reviewed literature to identify drivers. This project consisted of a bundle of interventions aimed at reducing the non–value-added time in the current process. Interventions included streamlining the triage process, a system of notification of all staff when these patients arrived in unit, a protocol that limited the length of time spent in attempting access to ports, nurse champions on every shift, and departmental awareness of progress by use of monthly scorecards. These interventions (Table 1) were begun in May 2016. During the active prospective evaluation phases, the ED staff reviewed data monthly and reviewed each case failing to meet the 1 hour goal in an effort to identify potential causes of delays in antibiotic administration.

**Table 1.**
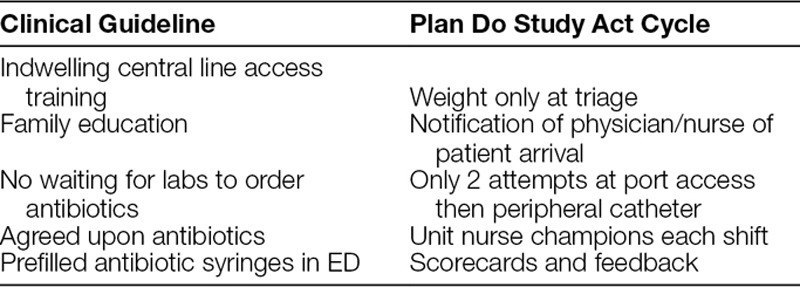
Interventions

### Analysis

Analysis of data was conducted by the principal investigator. The inclusion criteria were as follows: pediatric patients (birth to 18 years) with a chief complaint of fever (defined as any single temperature of ≥ 38.3°C or 2 measured temperatures between 38 and 38.2°C within a 12-hour period) and a history of having received chemotherapy within the past 6 months. Each patient visit that met the above criteria were evaluated through the electronic medical record. Software used to analyze trends was Minitab. Overall time to antibiotics and process measures of arrival to bed placement, bed placement to antibiotic order, and antibiotic order to delivery times were evaluated. Student’s *t* test was utilized to compare samples. This article was developed in accordance with Standards for Quality Improvement Reporting Excellence (SQUIRE 2.0) guidelines.^7^

### Measures for Improved Workflow

The primary variable was percentage of patients receiving antibiotics within 60 minutes of arrival. The following process metrics were evaluated during the improvement period:

- time from triage to ED room
- time from ED room to antibiotic order
- time from antibiotic order to antibiotic administration

Baseline data (before any intervention), time period 1 (after the initial guideline and order set but before the bundle of interventions discussed in this article) and time period 2 (after the bundle of interventions), were collected. Control charts were used to track data changes over time and to assess variation.

## RESULTS

**Fig. 1.**
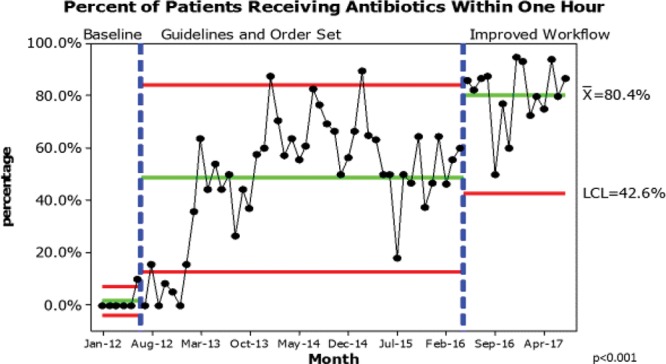
Percentage of patients receiving antibiotics within 1 hour.

Control charts revealed decreased variation and overall improved percentage of patients receiving antibiotics within 1 hour (Fig. 1). This improvement is both clinically and statistically significant (*P* < 0.001). Both the emphasis on improved workflow and availability of preferred antibiotic led to additional improvement, resulting in the percentage of patients receiving antibiotics within 1 hour rising to 80%. All variation during this improvement phase is common cause variation, with no evidence for special cause variation; however, our monthly performance showed a trend toward continued improvement, attributed to our real-time investigation into all cases that did not meet our 60 minutes goal.

**Fig. 2.**
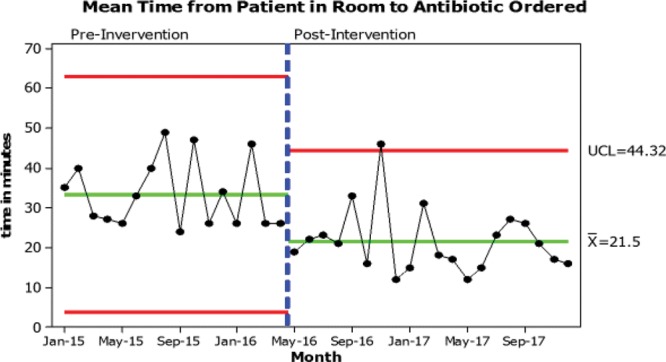
Mean time from patient in room to antibiotic ordered.

**Fig. 3.**
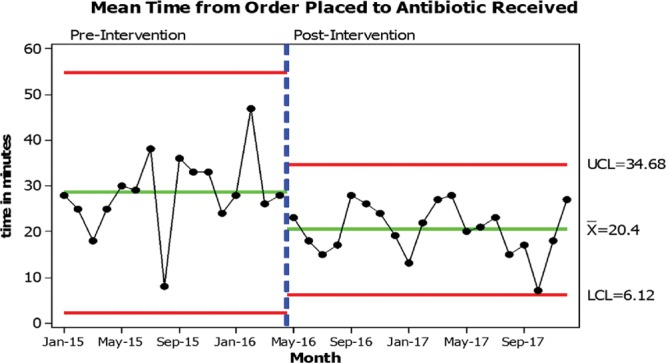
Mean time from order placed to antibiotic received.

Process measures were followed during our workflow improvement and can be seen in Figures 2, 3. The goal for the mean time from patient arrival to patient being placed in a room was less than 10 minutes. After our bundle of improvement, our average time to place a patient in a room increased slightly and displayed greater variation. Figure 2 shows the mean time from placement in room until antibiotic order placed with the goal of less than 25 minutes. On average, we exceeded this goal; however, some special cause variation existed. Figure 3 shows the mean time from antibiotic order until antibiotic administration was begun. The goal was less than 25 minutes; again on average we exceeded this goal. Although Figure 3 does not display special cause variation, there were still opportunities for improvement. The improvements in the times from the patient being placed in a room, to the antibiotic being ordered, and ultimately to antibiotic administration have improved with our new, streamlined workflow.

## CHALLENGES

During this improvement project, a shortage in our designated first-line antibiotic, cefepime, occurred in September 2016. This shortage was not anticipated, resulting initially in delays in timely antibiotic administration. The designated second choice antibiotic piperacillin/tazobactam is more complicated to order as it is a combination antibiotic, requiring additional time to mix. Nurses were spending up to 20 minutes mixing this medication. Our real-time investigation of changes in our process performance allowed us to identify this issue more quickly than we otherwise would have. The team reassembled to develop standard dosing regimens for which pharmacy could then premix doses for the ED. This has provided us with invaluable institutional knowledge as medication shortages will continue to be a challenge for health care delivery. We now prepare for medication shortages in initial order sets and have plans ready for implementation in future shortages.

An additional lesson learned was that instead of monthly scorecards, more immediate, just in time: feedback is preferred by frontline providers, so they may learn from signals in the process.

## DISCUSSION

Before any interventions, the baseline time to antibiotics was 63 minutes with only 30% of patients receiving antibiotics within the goal time of 1 hour. Following the initiation of the clinical guideline, the percentage of patients receiving antibiotics within 1 hour increased from 30% to 55%, and by the time we began our new, improved workflow, was averaging 48% with minimal special cause variation. This improvement project built on the momentum of the initial effort to achieve timely administration of antibiotics focusing more on process metrics to achieve improvement in each step of the process for an overall improvement of 80% of patients receiving antibiotics within the goal of 1 hour.

The strengths of the project include the use of process measures, which were invaluable to the success of this project. Small delays in each step of the patient’s path to antibiotics added up to make the overall goal difficult to achieve. For each barrier identified, possible solutions were devised and implemented. Lack of early recognition of immunocompromised state was leading to slower triage times. The pediatric oncology group reeducated families on the importance of clear communication upon arrival to the ED that their child had recently received chemotherapy. Patient care guidelines were created to instruct ED triage nurses to promptly place these potentially immunocompromised patients in a room without waiting on vital sign as triage vital signs do not influence patient care with regard to the decision to give antibiotics.

A second barrier identified was a lack of awareness by physicians and nurses that an oncology patient with fever had been placed in a room. Once a patient arrives, both physician and nurse need to act promptly. A key part of our bundled approach was the immediate notification of the physicians and nurses by ED communication devices of patient arrival prompting the initiation of the order set for labs and antibiotics and assess of the patient’s indwelling catheter.

Another common barrier identified was difficultly accessing the indwelling central lines or problems with the infusion pump. To address these barriers, the team instituted an algorithm designating prompt insertion of a peripheral intravenous catheter if the port-a-catheter was not easily accessed. Unit nurse champions were identified and provided key information for frontline staff surrounding the overall goal and the process metrics. Updates on data were provided to staff in daily shift huddles and e-mails. To improve staff engagement and understanding, “scorecards” were posted in staffing areas. The scorecards included current times for each of the process measures along with goal times for each. Individual care provider feedback was given to nurses and physicians in for patients experiencing prolonged time to antibiotic administration to better identify causes of delays and aid in ongoing improvement.

Our ability to manage preferred antibiotic shortages led to further improvement. During our new process implementation, our organization experienced a medication shortage of our preferred antibiotic for this patient population. Although this occurrence did not result in special cause variation, it was uncovered more quickly than would have been otherwise, because of our continual attention to process measures. Antibiotic shortages are certain to occur in the future, as are other barriers to improvement, such as staff turnover in an ED. Our experience in quick detection of a critical medication shortage has allowed us to be better prepared for future, similar challenges.

Sustainability of any QI project is always a challenge particularly in work areas with high turnover of staff. The scorecards were beneficial in keeping awareness of the goals high and in educating new staff about our specific goals. We have found this process to be useful and are extending the same QI process to other high risk groups of patients with fever who depend on timely administration of antibiotics. The clinical pathway and order set introduced previously assisted in improving the time to antibiotics; however, the assessment and improvement of our workflow at the point of care delivery was necessary to truly improve our practice.

An important point is our institution also utilizes a sepsis pathway. In cases of fever and high risk patients with positive sepsis screens, patients are diverted to the sepsis pathway in which fluids and antibiotics are emergently administered. This safe guard helps to ensure that fluid administration to potentially septic patients is not delayed.

## CONCLUSIONS

In patients who are immunocompromised, fever may be the first indication of a life-threatening infection.^8^ Recommended care for patients with neutropenia and fever is to administer broad spectrum antibiotics within 60 minutes of arrival to the ED. Achieving the goal of timely antibiotic administration in a pediatric ED can be difficult. Since the publication of the recommended guidelines for antibiotic administration in children with fever and neutropenia, many institutions have struggled to find ways to meet that recommendation within the ED setting. Clinical guidelines, order sets, and detailed understanding of the actual workflow at the point of care delivery can be instrumental in achieving the goals of reducing time to antibiotics. Using process measures for each step in the pathway can identify hidden delays and be the key to success.

## DISCLOSURE

The authors have no financial interest to declare in relation to the content of this article.
